# Supplementary material for: Curated and harmonised transcriptomics datasets of interstitial lung diseases
Source: Data Brief. 2025 Oct 14;63:112139. doi: 10.1016/j.dib.2025.112139 (PMC12581653; doi:10.1016/j.dib.2025.112139)
Supplement: Supplementary file 1 [file mmc1.zip › Supplementary_material/DNA-microarray/GSE10667/GSE10667_eUTOPIA_Agilent_QC_Report_2024-02-15.tar.gz!/Users/OWNER/AppData/Local/Temp/RtmpspbZX7/2024-02-15/hm.pdf]

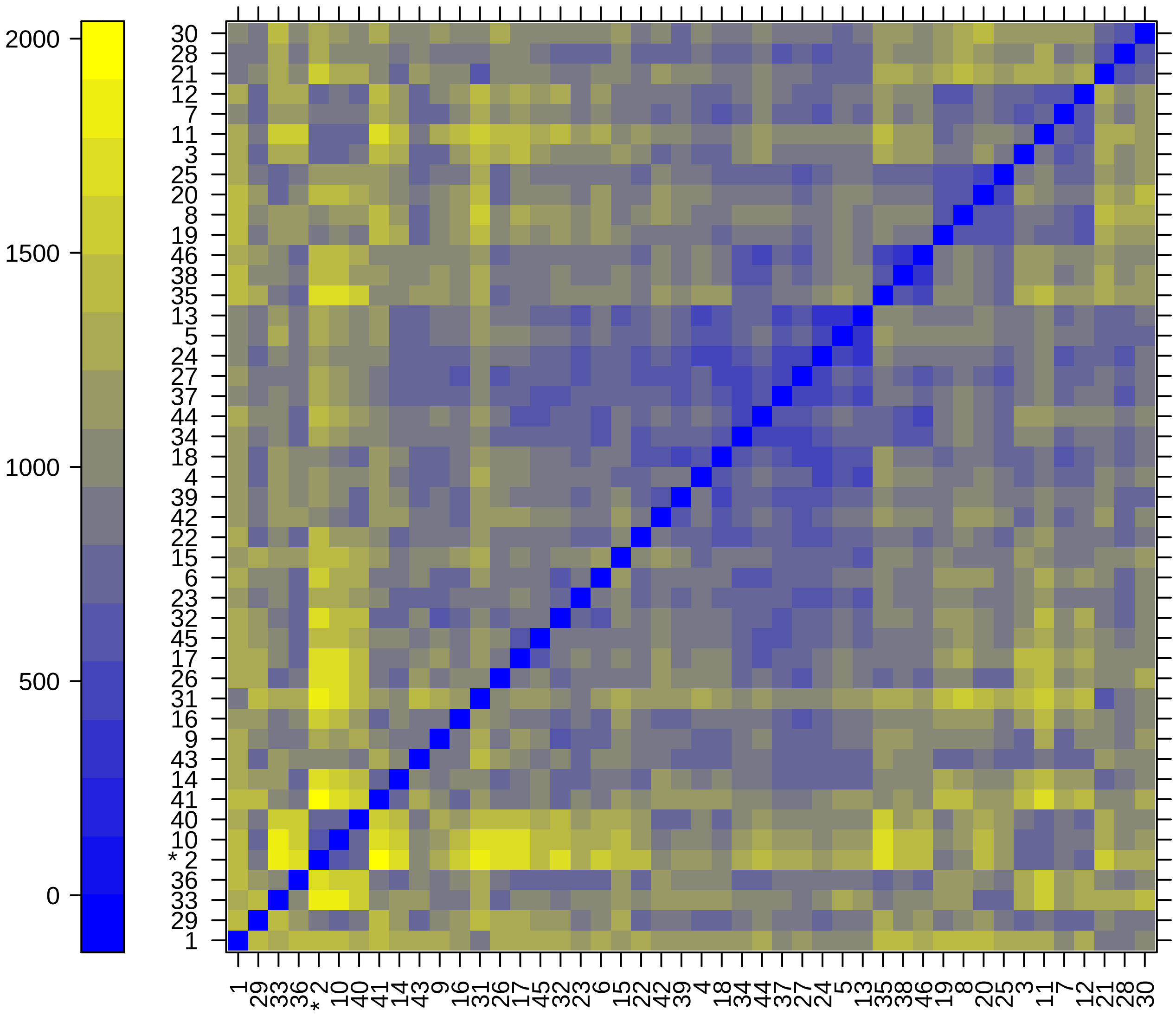

2000
1500
1000
500
0
30 28 21 12 7 11 3 25 20 8 19 46 38 35 13 5 24 27 37 44 34 18 4 39 42 22 15 6 23 32 45 17 26 31 16 9 43 14 41 40 10 *2 36 33 29 1
1 29 33 36 *2 10 40 41 14 43 9 16 31 26 17 45 32 23 6 15 22 42 39 4 18 34 44 37 27 24 5 13 35 38 46 19 8 20 25 3 11 7 12 21 28 30
